# Multidisciplinary Management of Heritable Aortopathy in Pregnancy Complicated by Postpartum Acute Type A Dissection

**DOI:** 10.1016/j.jaccas.2025.106234

**Published:** 2025-12-09

**Authors:** Alexandra E. Sperry, Aardra Rajendran, Michael Salna, Catherine Klammer, Grace J. Wang, Chase R. Brown, Lisa Levine, Jennifer Lewey, Nimesh D. Desai

**Affiliations:** a Division of Cardiothoracic Surgery, Department of Surgery, University of Pennsylvania, Philadelphia, Pennsylvania, USA; b Division of Cardiology, Department of Medicine, University of Pennsylvania Perelman School of Medicine, Perelman Center for Advanced Medicine, Philadelphia, Pennsylvania, USA; c Department of Cardiothoracic Surgery, New York University, New York, New York, USA; d Division of Maternal-Fetal Medicine, Department of Obstetrics and Gynecology, Perelman School of Medicine, University of Pennsylvania, Philadelphia, Pennsylvania, USA; e Division of Vascular Surgery Department of Surgery, University of Pennsylvania, Philadelphia, Pennsylvania, USA

**Keywords:** aortic dissection, connective tissue disease, genetic syndrome, hereditary thoracic aortic disease, pregnancy

## Abstract

**Case Summary:**

A 33-year-old patient at 20 weeks of gestation presented with a dilated aortic root and abdominal aorta, and a diagnosis of Loeys-Dietz syndrome was made.

**Key Questions:**

What is the incidence of pregnancy-related aortic dissection in patients with heritable thoracic aortic disease (HTAD)? What are the key management principles for pregnant patients with HTAD?

**Outcome:**

A shared plan was made by a multidisciplinary team of maternal fetal medicine, cardio-obstetrics, and cardiothoracic surgery, consisting of imaging surveillance, blood pressure control, scheduled cesarean delivery, and future elective aortic surgery. After an uncomplicated delivery, the patient experienced an acute type A dissection requiring staged total aortic replacement, including aortic root and arch replacement, thoracoabdominal aortic replacement, and endovascular aortic repair.

**Take-Home Messages:**

Patients with HTAD are at an increased risk of pregnancy-related aortic dissection. Key management principles include multidisciplinary evaluation, imaging surveillance, blood pressure control, and delivery with cardiothoracic surgical backup.

## Visual Summary

**Figure:**
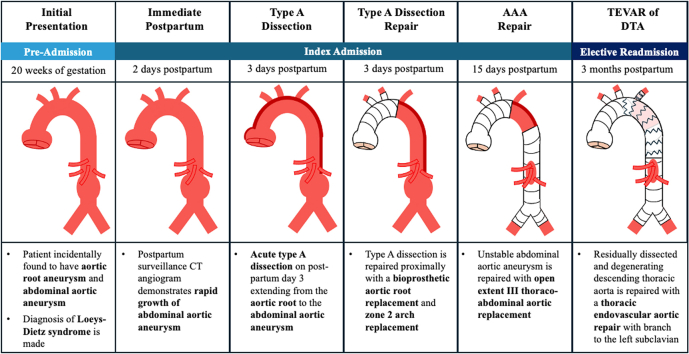

## Case Presentation

**Figure 1:**
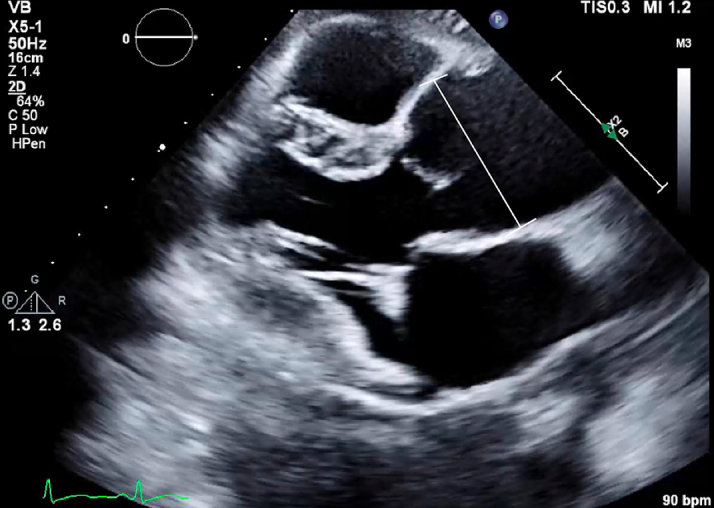
Preoperative Transthoracic Echocardiogram Demonstrating a Dilated Aortic Root to 4.1 cm

A 33-year-old pregnant patient (G1P0) with no significant medical history presented for routine 20-week prenatal ultrasound, and a dilated infrarenal aorta at 3.8 cm was incidentally discovered. Transthoracic echocardiography (TTE) demonstrated a 4.1-cm aortic root aneurysm (Figure 1). Given the need for complex multidisciplinary evaluation, she was referred from her community hospital to an academic institution.

Outpatient care was initiated with the maternal fetal medicine (MFM) and cardio-obstetrics teams. At initial consultation, the patient was 25 weeks pregnant and without cardiovascular symptoms. Physical examination did not identify manifestations of connective tissue disease, although careful review of family history revealed ruptured cerebral aneurysm in the patient's mother; family history was otherwise negative for aortic disease. Given the collective findings, consultation with medical genetics to evaluate for heritable thoracic aortic disease (HTAD) was recommended; furthermore, the decision was made to pursue cross-sectional imaging of the aorta and cerebral vasculature with magnetic resonance angiography of the chest and abdomen without contrast, and computed tomography angiography (CTA) of the brain with intravenous contrast, which were completed at approximately 32 weeks.

**Figure 2:**
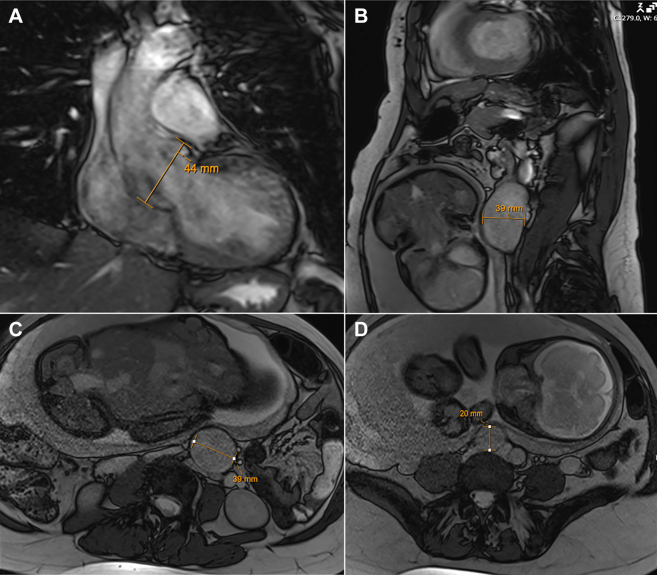
Preoperative MRI of the Chest, Abdomen, and Pelvis Preoperative MRI demonstrating (A) 4.4-cm aortic root, (B and C) 3.9-cm infrarenal abdominal aortic aneurysm, and (D) 2.0-cm right common iliac artery aneurysm, characteristic of the diffuse aneurysmal disease seen in Loeys-Dietz syndrome. MRI = magnetic resonance imaging.

Genetic testing demonstrated a pathogenic variant in the *SMAD3* gene, consistent with Loeys-Dietz syndrome (LDS) type III. Imaging revealed a 4.4-cm aortic root, a 3.9-cm infrarenal abdominal aortic aneurysm (Video 1), a 2.0-cm right common iliac aneurysm, and no intracranial findings (Figure 2); thus, the patient was referred to cardiothoracic surgery. A long discussion was held with the patient regarding LDS and risk of type A aortic dissection in the peripartum period given aortic root dilatation. Risks and benefits regarding mode of delivery were explained to her, with options for either vaginal delivery with assisted second stage or planned cesarean delivery. A comprehensive care plan for the remainder of the pregnancy, delivery, and postpartum period was made between the patient and the team of MFM, cardio-obstetrics, and cardiothoracic surgery, which included continued surveillance throughout the pregnancy by all teams, anti-impulse control with beta-blockade, elective cesarean delivery at 39 weeks, immediate CTA after delivery, and elective aortic root replacement in the following year.

The remainder of the patient's pregnancy was uncomplicated, and her blood pressure was maintained at <120/80 mm Hg with heart rate <80 beats/min on 25 mg metoprolol daily. Repeat surveillance TTE was performed in the third trimester, 12 weeks after the initial echocardiogram, and demonstrated a stable root. Fetal TTE demonstrated no findings suggestive of LDS.

**Figure 3:**
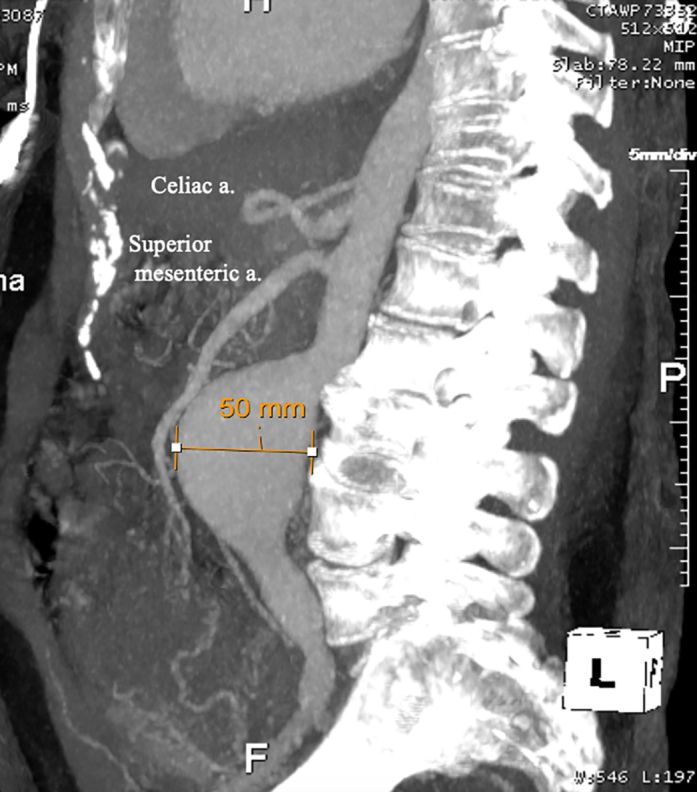
CTA on Postpartum Day 2 Demonstrating Rapid Growth of the Infrarenal Aorta to 5.0 cm CTA = computed tomography angiography.

**Figure 4:**
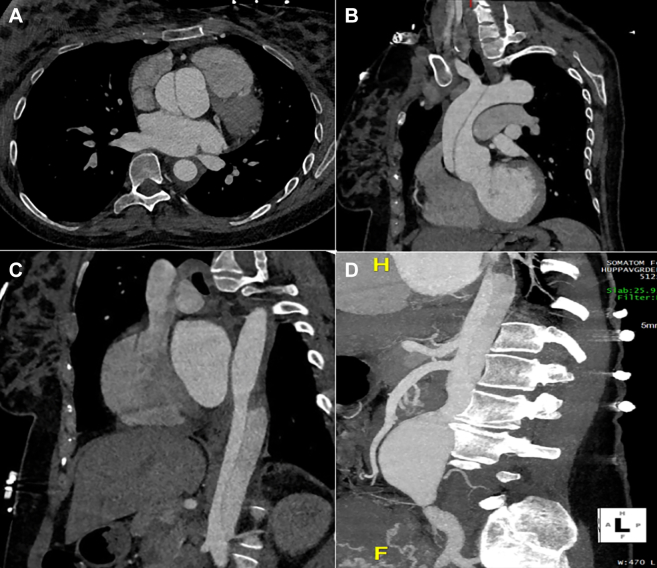
CTA on Postpartum Day 3 Demonstrating New Debakey I Aortic Dissection (A and B) The aortic dissection originated at the level of the aortic root, and (C and D) it extended distally to the infrarenal abdominal aortic aneurysm. CTA = computed tomography angiography.

The patient was electively admitted at 39 weeks for planned cesarean; blood pressure was well-controlled throughout, and she delivered a healthy baby girl without complications. CTA performed on postpartum day 2 demonstrated rapid growth of the infrarenal abdominal aortic aneurysm to 5.0 cm without changes to the ascending aorta or root (Figure 3). Both vascular and cardiothoracic surgery were consulted, and a plan was made for open repair. However, the following day, the patient experienced sudden chest and back pain, and emergent CTA demonstrated new Debakey I aortic dissection (Figure 4). She was taken emergently to the operating room with cardiothoracic surgery for proximal repair, where inspection revealed a large primary tear at the sinotubular junction and a second tear between the innominate and left common carotid arteries; thus, a bioprosthetic root replacement and zone 2 arch replacement were performed (cardiopulmonary bypass: 169 minutes; cross-clamp: 149 minutes; circulatory arrest with antegrade cerebral perfusion: 51 minutes). Left ventricular ejection fraction (LVEF) remained unchanged at 55% before and immediately after repair.

The patient's postoperative course was uneventful, and surveillance CTA demonstrated intact proximal repair and unchanged thoracoabdominal aorta. Given her LDS diagnosis, the rapid growth of the infrarenal aneurysm, and the multiple complex dissection flaps within the thoracoabdominal aorta, the decision was made between cardiothoracic and vascular surgery to intervene with open surgery. On postpartum day 15 (postoperative day 12), the patient was taken for open extent III thoracoabdominal aortic aneurysm repair with bifurcated graft to the iliac arteries. Recovery was once again uneventful, and she was discharged home 24 days after the index proximal repair (postpartum day 27), with full neurologic function.

The patient was seen in clinic 1 month after discharge, and surveillance CTA demonstrated degeneration of the residually dissected native proximal descending thoracic aorta between the surgical grafts (Video 2), so elective thoracic endovascular aortic repair was recommended. Notably, TTE at this time demonstrated a new reduction in LVEF to 25%, with ventricular dilation (left ventricular internal dimension at diastole: 6.2 cm), so guideline-directed medical therapy for heart failure was initiated. However, the patient subsequently developed nausea and poor oral intake, and was admitted at 2 months postpartum with TTE demonstrating further reduction in LVEF to 5% to 10%. Coronary angiography was normal, but cardiac magnetic resonance imaging indicated diffuse interstitial fibrosis without late gadolinium enhancement, consistent with *SMAD3* cardiomyopathy, exacerbated by postpartum and surgical stress. She was successfully managed with guideline-directed medical therapy and optimized for surgery.

**Figure 5:**
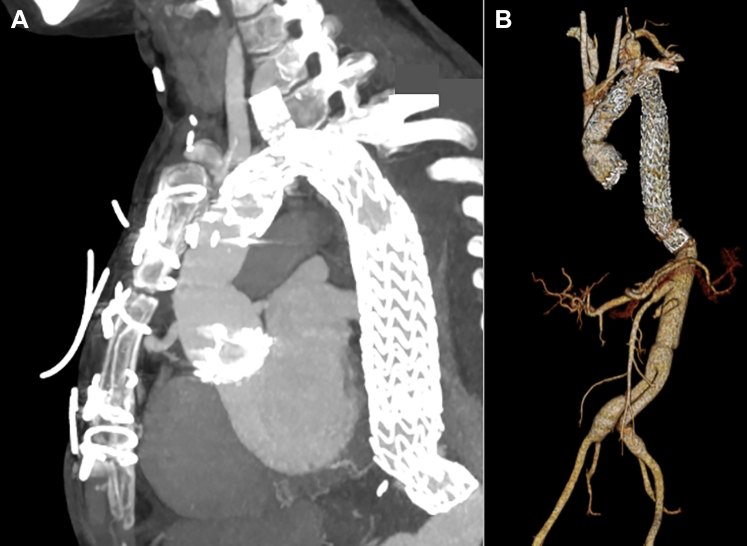
Postoperative Surveillance CTA After TEVAR Postoperative CTA demonstrated (A) thoracic branched endograft without endoleak and (B) complete aortic replacement from the aortic root to the bilateral iliac arteries. CTA = computed tomography angiography; TEVAR = thoracic endovascular aortic repair.

Three months after the index proximal repair, the patient underwent a thoracic branched endograft from zone 2 (distal to the left common carotid artery) to zone 5 (proximal to the celiac artery), with branch into the left subclavian artery. Surveillance CTA demonstrated intact complete aortic replacement from the level of the aortic root to the celiac artery, with excellent aortic remodeling with false lumen thrombosis (Figure 5, Video 3). The patient has done well since this admission, and recent TTE demonstrated improved LVEF to 35%. She is symptomatically doing well, able to engage in regular exercise, independently perform all activities of daily living, and care for her baby daughter.

## Key Questions

### Question 1: How does HTAD affect the risk of pregnancy-related aortic dissection?

HTAD is a family of genetic conditions caused by mutations in specific genes that, when altered, confer a highly penetrant risk for aortic aneurysm and dissection.^1^^,^^2^ Given the rarity of inherited aortopathies, most evidence regarding the natural history of pregnancy in this population is based on small prospective registries and retrospective cohort studies; however, these studies have consistently demonstrated a strong association between HTAD and pregnancy-related aortic complications.

A study from the International Registry of Aortic Dissection found that 69% of pregnancy-related aortic dissections occurred in those with HTAD.^3^ The estimated incidence of pregnancy-related aortic complications in the HTAD population varies by study and by pathogenic variant: Results from the GenTAC Registry demonstrated that among pregnant women with Marfan syndrome, 10.6% experienced an aortic complication, while the prospective global Registry of Pregnancy and Cardiac Disease (ROPAC) III found a 3.5% incidence of pregnancy-related aortic dissection across a heterogeneous HTAD group.^4^, ^5^, ^6^ LDS is traditionally under-represented in these studies, with most cases of HTAD being among those with Marfan syndrome; therefore, the individual risks with LDS are less well described. Regardless, the reported incidence of aortic complications in HTAD patients is dramatically higher than that of the overall population (0.0012%).^6^ Furthermore, while pregnancy is a known risk factor for aortic complications for all patients, conferring a 2.86-fold increase in adverse aortic events, this risk is markedly higher in the HTAD population, with an estimated 5- to 8-fold increase in aortic complications compared with the nonpregnancy period.^5^^,^^6^

### Question 2: For patients with HTAD who are pregnant or considering future pregnancy, what are the key principles of preconception and antepartum management?

For patients with HTAD contemplating pregnancy, the first key principle is to involve a multidisciplinary team that includes MFM and cardiology, and ensure the patient is included in shared decision-making conversations.^7^, ^8^, ^9^ Preconception considerations that should be discussed include the risks and implications of pregnancy, specifically the significantly increased risk in maternal morbidity and mortality when a dilated aorta is present, as well as genetic counseling to discuss the heritable nature of the aortopathy, imaging of the aorta for risk assessment, and the need for prophylactic aortic surgery. Recommended imaging modalities include transthoracic echocardiography for assessment of the aortic root, and magnetic resonance angiography with contrast or CTA for assessment of the aortic arch, descending thoracic aorta, and abdominal aorta. Additional imaging may be required based on pathogenic variant.^7^

If the patient becomes pregnant, initiation of beta-blocker therapy is recommended, with or without a diagnosis of hypertension, to lessen hemodynamic stress on the aorta by controlling blood pressure and heart rate.^7^^,^^8^ Additionally, surveillance imaging is recommended for both the proximal and distal aorta; proximally, TTE should be performed each trimester, and again postpartum, to monitor the aortic root and ascending aorta for growth. Distally, while monitoring of the aortic arch, descending, and abdominal aorta with magnetic resonance imaging without gadolinium is optimal to avoid radiation exposure to the fetus, it is reasonable to consider CTA in certain high-risk clinical situations, as the radiation exposure would still be below the threshold for concern.^7^^,^^8^ If aortic dilation is present, cardiothoracic surgery should be engaged regarding a contingency plan for emergency aortic repair.

### Question 3: For patients with HTAD, what are the thresholds for prophylactic surgical intervention on the aortic root and ascending aorta outside of pregnancy?

Hereditary aortopathies represent a diverse spectrum of diseases with varying rates, risks, and presentations of aortic complications; thus, there is no single threshold for prophylactic intervention in these patients.^1^^,^^2^ For patients with Marfan syndrome, prophylactic aortic surgery is recommended when the aortic root or ascending aorta has reached a diameter of at least 5.0 cm, or 4.5 cm in the setting of high-risk features. Alternatively, for LDS patients, surgical recommendation depends on the causal pathogenic variant; those with the more aggressive *TGFBR1*, *TGFBR2*, and *SMAD3* variants can be recommended surgery at diameters as low as 4.5 cm.^7^ In general, for patients with HTAD and aortic dilatation, the threshold for prophylactic surgical intervention should be informed by the individual patient risk profile, which includes factors such as the specific genetic variant, the aortic diameter and growth rate, systemic phenotypic features, and family history, along with patient preference.

### Question 4: For patients with HTAD, how does pregnancy affect the threshold for prophylactic surgical intervention?

Specific evidence regarding aortic diameters and dissection risk related to pregnancy in HTAD is scarce, as most pregnancy-related dissections occur in women previously unaware of an HTAD diagnosis.^10^ The cardiovascular and hormonal physiologic changes of pregnancy lead to increases in maternal blood volume, heart rate, stroke volume, and cardiac output, which in turn increase the risk of aortic complications, particularly in the third trimester and through 12 weeks postpartum; therefore, preconception thresholds for surgical prophylaxis tend to be at least 0.5 cm lower than those for nonpregnant individuals.^3^^,^^7^^,^^8^ Notably, while guidelines focus on interventions for a the proximal aorta, there are few overt recommendations on thresholds for intervention on the descending thoracic or abdominal aorta.

Management is more complex in patients who are already pregnant when they receive a diagnosis of HTAD with aortic dilatation and requires individualized decision-making that balances the risks of maternal aortic dissection or rupture with the perioperative burden of cardiac surgery to the fetus. If a patient is deemed high risk owing to aortic size or rapid growth and is early in the pregnancy, prophylactic surgery should be considered, versus a combined delivery and repair if later in the pregnancy.^8^ Notably, fetal mortality in patients undergoing aortic surgery requiring cardiopulmonary bypass may be as high as 30%.^7^, ^8^, ^9^ Thus, if a woman is deemed high risk early in the pregnancy, she may pursue termination to facilitate subsequent prophylactic aortic repair.

### Question 5: What are the considerations for mode of delivery in patients with HTAD?

Guidelines regarding optimal timing and strategy of delivery for women with HTAD and thoracic aortic disease are primarily based on expert opinion and limited observational data. Firstly, it is recommended that women with any history of aortopathy deliver at a specialized center with a pregnancy heart team and on-call cardiothoracic surgery.^7^, ^8^, ^9^ Regarding mode of delivery, recommendations favor a vaginal delivery when aortic diameter is <4.0 cm, caesarean delivery when >4.5 cm, and either caesarean or vaginal delivery with regional anesthesia and assisted second-stage labor based on patient preference when between 4.0 and 4.5 cm.^7^^,^^8^ Timing of delivery is usually planned for 37 to 39 weeks to decrease the risk of hypertensive disorders of pregnancy, although there is limited evidence to support this.^9^ Finally, a predetermined contingency plan with cardiothoracic surgery in the case of peripartum aortic complication is necessary to facilitate expeditious surgical intervention.

**Visual Summary:**
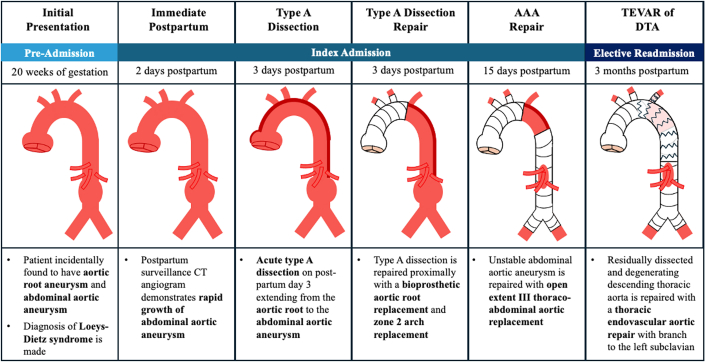
Stages of Total Aortic Replacement in a Patient With HTAD After Postpartum Aneurysm Growth and Type A Aortic Dissection AAA = abdominal aortic aneurysm; CT = computed tomography; DTA = descending thoracic aorta; TEVAR = thoracic endovascular aortic repair.

## Funding Support and Author Disclosures

Dr Sperry has received support from a T32 10.13039/100000052NIH Grant (T32 HL00784). Dr Desai has been a consultant for Gore Medical, Terumo Aortic, and Edwards Lifesciences. All other authors have reported that they have no relationships relevant to the contents of this paper to disclose.

**Take-Home Messages**

• Women with heritable thoracic aortic disease are at an increased risk of pregnancy-related aortic dissection, and frequent imaging surveillance of the entire aorta before and after delivery is necessary to both understand baseline risk and monitor disease progression.
• For women with genetic aortopathy, shared multidisciplinary evaluation between maternal fetal medicine, cardio-obstetrics, and cardiothoracic surgery throughout the pregnancy facilitates patient education, blood pressure control, monitoring for complications, and safe delivery with urgent surgical backup if needed.

## Appendix

Video 1 Preoperative MRI of the Abdomen and Pelvis Demonstrating a 3.9-cm Infrarenal Abdominal Aortic Aneurysm.

Video 2 Postdischarge Surveillance CTA CTA demonstrated aneurysmal degeneration of the residually dissected aorta between the grafts in zone 2 and in the distal descending thoracic aorta, necessitating intervention with TEVAR. CTA = computed tomography angiography; TEVAR = thoracic endovascular aortic repair.

Video 3 Surveillance CTA After TEVAR With Thoracic Branched Endograft CTA demonstrated intact proximal and distal landing zones with successful aortic remodeling and false lumen thrombosis. CTA = computed tomography angiography; TEVAR = thoracic endovascular aortic repair.
